# Survival based radiographic-grouping for esophageal squamous cell carcinoma may impact clinical T stage

**DOI:** 10.18632/oncotarget.24056

**Published:** 2018-01-09

**Authors:** Wenjie Cai, Jiade J. Lu, Rongyu Xu, Peiling Xin, Jun Xin, Yayun Chen, Bingzhong Gao, Jieyun Chen, Xiyang Yang

**Affiliations:** ^1^ Department of Radiation Oncology, First Hospital of Quanzhou Affiliated to Fujian Medical University, Quanzhou 362000, P. R. China; ^2^ Shanghai Proton and Heavy Ion Center, Shanghai 201315, P. R. China; ^3^ Department of Surgical Oncology, First Hospital of Quanzhou Affiliated to Fujian Medical University, Quanzhou 362000, P. R. China; ^4^ Department of Surgery, First Hospital of Quanzhou Affiliated to Fujian Medical University, Quanzhou 362000, P. R. China; ^5^ Department of Radiology, First Hospital of Quanzhou Affiliated to Fujian Medical University, Quanzhou 362000, P. R. China; ^6^ Key Laboratory of Intelligent Computing and Information Processing, Quanzhou Normal University, Quanzhou 362000, P. R. China

**Keywords:** esophageal cancer, tumor staging, CT, imaging, prognosis

## Abstract

Most patients diagnosed with thoracic esophageal squamous cell carcinoma (ESCC) have progressed beyond surgical resection as a therapeutic option. Difficulties in the proper assessment of tumor invasion depth before treatment complicate determination of the type and extent of therapy. Therefore, accurate tumor clinical staging is a necessity for identifying treatment options and aiding in patient prognosis. We investigated radiographic factors as prognostic indicators for survival in ESCC. Between July 2006 - July 2010, 324 thoracic ESCC patients who underwent surgery were selected. All patients received contrast enhanced preoperative chest CT scans and esophageal barium swallow examinations. Measurement of maximal lesion cross-sectional area, the largest long diameter, largest short diameter, CT-indicated lesion length, barium-indicated lesion length and the length of pericardial fat reduction were performed. Relationships between these indicators and post-surgical survival time and the cutoff values of related factors were analyzed. Maximum long diameter, maximum lesion area and lesion length, as measured by CT imaging, were correlated with survival. Survival effects were clearly associated with group intervals, calculated by a genetic algorithm, and tumor stages. Risk-stratification intervals of esophageal lesions from radiographic imaging included: maximum long diameter < 28.7, 28.7-34.6mm, 34.6-41.4mm and >41.4mm; maximum lesion area < 355.8mm^2^, 355.8-568.0mm^2^, 568.0-907.3mm^2^ and >907.3mm^2^; and CT-indicated lesion length <30.9mm, 30.9-57.3mm, 57.3-70.6mm and > 70.6mm. The reasonable stratification of maximum esophageal lesion area, largest long diameter and lesion length measured in CT is valuable for clinical T staging of ESCC. Radiographic parameters may have prognostic clinical value in the staging of esophageal carcinoma.

## INTRODUCTION

Esophageal squamous cell carcinoma (ESCC) is a global health concern, and prevalence is especially high in certain populations in China [1]. The vast majority of patients diagnosed with ESCC enter the clinic at mid- to late stages of disease, and approximately 60% of those individuals have progressed beyond surgical resection as a therapeutic option. These statistics are striking, considering many patients exhibit no symptoms upon diagnosis [2]. Unfortunately, most patients with esophageal carcinoma die within the first 5 years following diagnosis [3]. As such, non-surgical modalities of treatment are of great importance for this population of ESCC patients. Unfortunately, difficulties in the proper assessment of tumor invasion depth before treatment complicate determination of the type and extent of therapy for these patients. Therefore, accurate tumor clinical staging is a necessity for identifying treatment options and aiding in patient prognosis.

The TNM Classification of Malignant Tumors (TNM) system developed by the Union for International Cancer Control (UICC) and the American Joint Committee on Cancer (AJCC) is widely used for tumor staging; however, it currently is only applied in cases of surgical transection in esophageal cancer cases. The depth of tumor invasion determines the T stage in the TNM system, not tumor size or other tumor burden metrics. Endoscopic ultrasound (EUS) is a commonly utilized imaging method for diagnosis and staging of ESCC prior to any treatment. Its utility lies in the ability to differentiate esophageal wall layers. However, the value of EUS for T staging is somewhat unclear due to variability in clinical reports. Though some studies report that ultrasound is useful in identifying early stages of ESCC [4, 5], others suggest is has more diagnostic efficacy in advanced stages of disease [6]. In addition, EUS appears to be less specific and sensitive in determining the depth of tumors, and thus T staging, near the gastroesophageal junction [7]. This is problematic as the majority of esophageal carcinomas occur in the lower esophagus. Other methods of imaging may exhibit a better ability to stage tumors earlier in patients.

In developing countries, including China, EUS and PET / CT clinical staging has not been widely used as a result of limiting economic factors. Starting in 1977, non-surgical staging using barium swallow-indicated esophageal lesion length as staging criteria continues to be widely utilized. However, barium swallow assessment is a dynamic imaging test never clinically intended to accurately provide size and dimension information of esophageal tumors. The consistency of results for this test are dependent on variable patient factors and the radiology personnel performing the tests, making reliability of the barium method questionable.

Computed tomography (CT) scanning is among the most widely used imaging tools for tumor staging prior to surgery. Benefits of CT imaging include the clarity and specificity that allows visualization of esophageal wall thickness and anatomical perimeters. Such detailed imaging is a limitation of EUS compared to CT imaging for the purposes of tumor staging. A clear understanding of tumor depth, as well as extent of local and widespread metastasis, are crucial for developing treatment approaches [8]. Although EUS is often used in assessing tumor depth and initial tumor staging [9], CT imaging is more effective at visualizing the 3-dimensional extent of tumors, their invasion into local structures and involvement of lymph nodes [10]. In addition, CT imaging also provides useful data to radiation oncologists for determining therapy volume. Considering that many clinical centers, EUS and PET are not routinely used for diagnosis and staging of esophageal carcinoma due to shortage of equipment and funding, we prefer to more widespread use of CT imaging for esophageal tumor staging, treatment planning, and prognostication.

For the current study, morphological characteristics of tumor lesions and associated regional anatomical changes were analyzed from 324 patients with ESCC who underwent surgical resection. Imaging modalities and gross examination of tumor tissues were performed and patient survival time was assessed. In summary, the goal of this study was to improve the clinical utility of T staging for esophageal squamous cell carcinoma.

## MATERIALS AND METHODS

### Ethics statement

Study participants voluntarily agreed to participate in the study and provided written informed consent prior to enrollment. The study was approved by the Ethics Committee of First Hospital of Quanzhou Affiliated to Fujian Medical University. All procedures performed in studies involving human participants were in accordance with the ethical standards of the institutional and/or national research committee and with the 1964 Helsinki declaration and its later amendments or comparable ethical standards.

### Clinical data

We obtained esophageal cancer patient information from medical records of the First Hospital of Quanzhou Affiliated to Fujian Medical University from July 2006 - July 2010. In this study, a total of 324 esophageal cancer patients underwent either oncologic or thoracic surgery. All patients received a preoperative, contrast-enhanced chest CT scan and 281 individuals underwent barium esophagraphy performed in our hospital.

We recommend that patients should be followed up every 3 months during the first year after surgery. Specific information should be collected and recorded including detailed medical history, and results of a physical examination. Upper gastrointestinal imaging, upper GI endoscopy, chest and/or abdomen CT, and neck/abdominal color Doppler ultrasound examination should be selected based upon the patient’s condition. Patient survival can be confirmed by monthly telephone follow-up, combined with household registration information.

### CT imaging and image analysis

For CT imaging (GE 64-slice spiral CT scanner), the scanning parameters were as follows: 120 kv, 90 mAs, collimation 5.0mm, pitch 25mm, bed-speed 50mm/s, thickness 10mm, layer interval 10mm. Scanning was performed from the neck down to the level of the hepatic portal. Patients received a cubital vein injection of nonionic iodinated contrast agent (100 ml, 3ml/s, 30s) prior to scanning. The raw scanning data were reconstructed to present an image of 2.5mm thickness.

**Figure 1:**
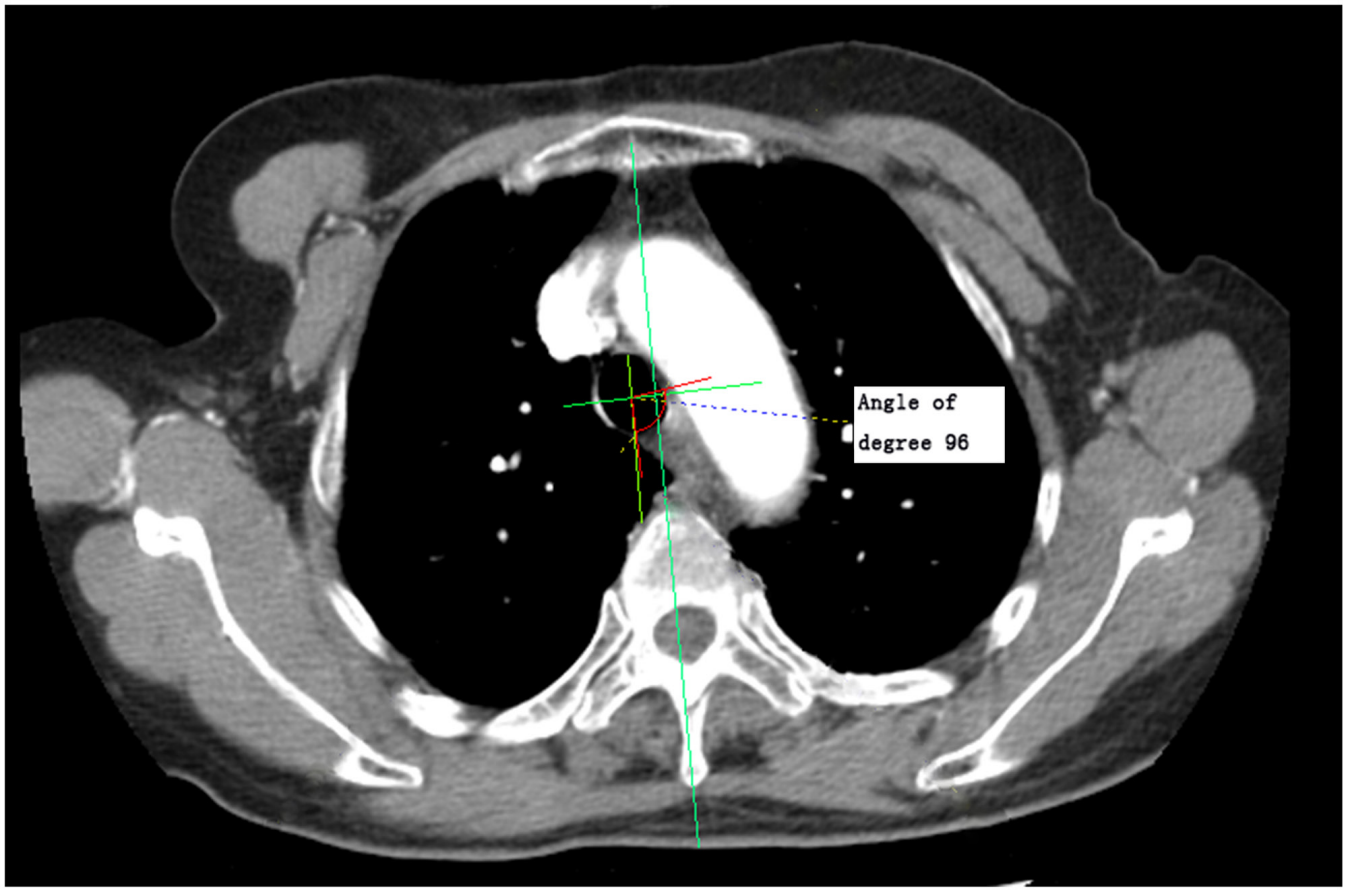
Measurement diagram of the maximum curvature formed by esophageal lesion contact with the trachea

**Figure 2:**
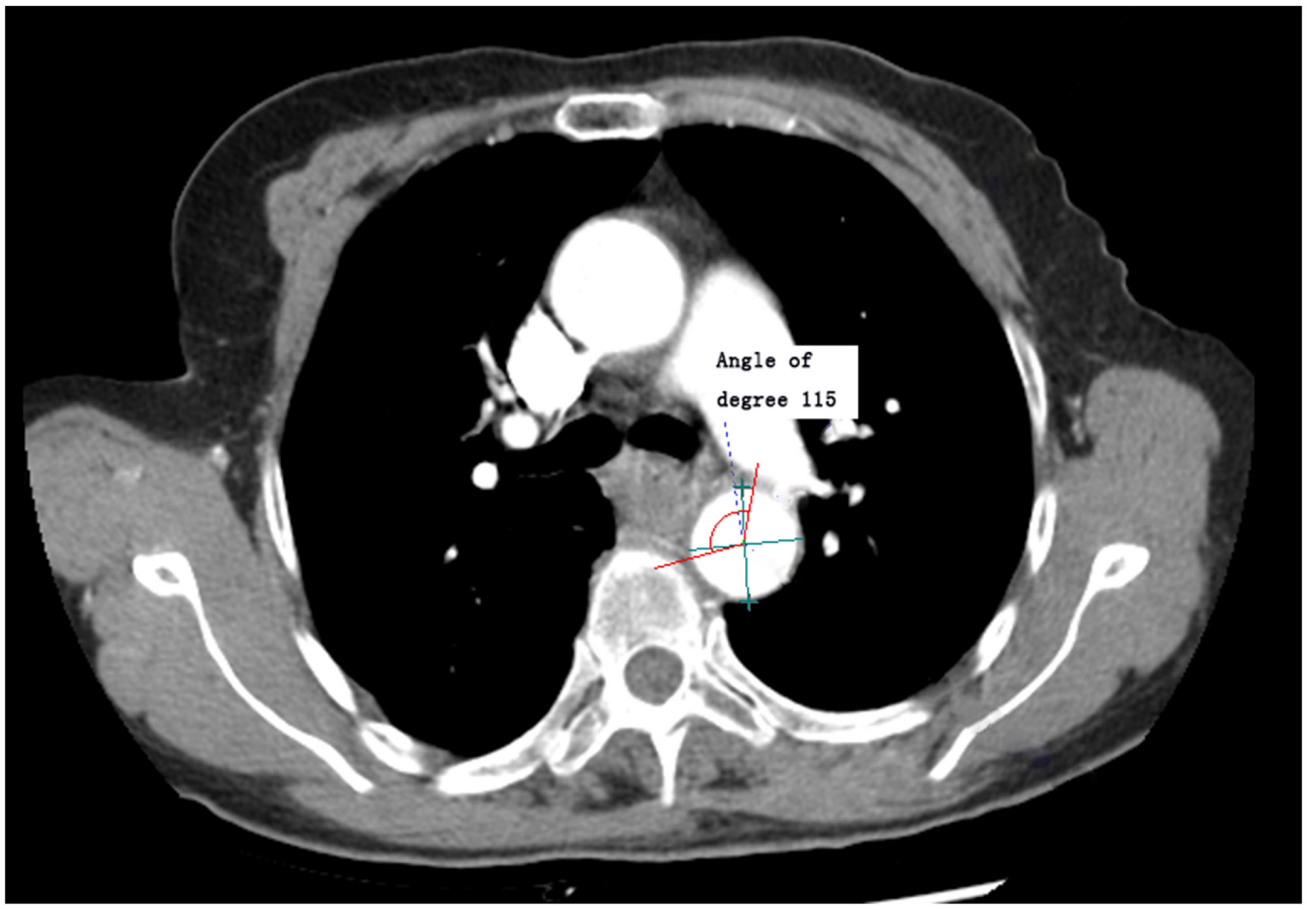
Measurement diagram of the maximum curvature formed by esophageal lesion contact with the thoracic aorta

In the NeusoftPacs 3.0 software, the reconstructed image was measured along CT mediastinal window according to the following standards and procedures: 1) Standards for defining presence of lesions were esophageal wall thickness > 5mm and esophageal diameter (without gas) > 10mm accompanied by local irregular luminal narrowing [11, 12]. 2) Visualization is performed in the layer in which the largest cross-sectional area of esophageal lesions is located. The image included the five layers above and below the largest cross-sectional layer, and the software outlined the cross-section, automatically calculated the cross-sectional area, and selected the maximum cross-sectional area. 3) According to the guidelines for evaluating solid tumor treatment response, the largest long diameter and the largest short diameter were measured [13]. 4) The vertical bisector method was used to obtain the center of the trachea/thoracic aorta. The central angle of the esophageal lesion and tracheal/thoracic aorta contact arc were measured using the software (Figures 1 & 2). 5) We calculated the total length of fat decrease between esophageal lesions and the pericardium.

### Examination following barium intake

Following barium swallow intake (130% W/V), the esophageal outline showing various filling states, mucosal fold characteristics, motility and density were visualized from multiple angles using an Italy GMM OPERA 800mA (DSA) multifunction digital X-ray machine. Anteroposterior, right anterior oblique and left anterior oblique films of the esophagus during barium ingestion were acquired, from which lesion lengths were measured.

### Imaging parameter measurements

The imaging parameters included: 1) maximal lesion cross-sectional area; 2) the largest long diameter; 3) the largest short diameter; 4) CT-indicated lesion length; 5) barium-indicated lesion length; 6&7) the curvature formed by lesion contact with the trachea and thoracic aorta, and, 8) the length of pericardial fat reduction, were independently measured by two experienced physicians, a radiologist and radiation oncologist, blinded to other clinical data. If the difference between the measured values by the two experienced physicians was less than 10%, the resulting measurement was the average of the two values. If the measurement differences between these two experienced physicians was greater than 10%, the chief physician in the radiotherapy department re-measured the data, then selected the prior value closest to the new measurement to produce an average.

### Statistical analysis

Statistical analyses were performed using SPSS 17.0 software. A paired Student’s t-test was used for the comparison of barium and CT-indicated lesion length values. Correlations between survival time and quantitative data were determined using Cox-regression analysis.

The differences in survival time among the groups were analyzed. All cases were sorted according to the date of operation, with odd cases selected as a training set, and even cases used as validation sets. For the training set, tumor characteristic grouping threshold *C_1_, C_2_, C_3_* were selected using a genetic algorithm (GA) for adequate separation of survival times between the four groups. The full dataset was assumed to have a mean μ, and the *i*th group exhibited a mean μ_i_. The separation of survival times in these four groups was primarily defined as the ratio of variance between groups to the variance within groups:

$$\underset{c_{1},c_{2},c_{3}}{\text{max}}0.9\frac{\sigma_{\text{between}}^{2}}{\sigma_{\text{within}}^{2}}+0.1\text{Var}({\text{Num}}_{1},\cdots ,{\text{Num}}_{4})$$ (1)

where

$$\begin{array}{c} \sigma_{\text{between}}^{2}=\frac{1}{4}\overset{4}{\underset{i=1}{\sum }}\underset{x_{j}\in \;{\text{Group}}_{\text{i}}}{\sum }{\left(\mu_{i}-\mu \right)}^{2},\;\sigma_{\text{within}}^{2}\\ =\frac{1}{4}\overset{4}{\underset{i=1}{\sum }}\underset{x_{j}\in \;{\text{Group}}_{\text{i}}}{\sum }{\left(x_{j}-\mu_{i}\right)}^{2}, \end{array}$$ (2)

and Var(*Num_1_, …, Num_4_*), the sample size variance of these 4 groups, helps avoid generating insufficiently small groups.

Maximal survival time separation occurs when tumor characteristic group points are appropriately selected. Grouping threshold in Equation (1) were optimized using the global optimization toolbox in Matlab. Survival analysis of surgical staging and different radiographic parameters was performed using Kaplan-Meier survival and Mantel-Cox log rank analyses. Consistency between staging approaches was analyzed with kappa statistics. A *P* value <0.05 was considered statistically significant.

## RESULTS

Of the 324 patients, 239 (73.8%) were male, 85 female (26.2%), with a median age of 56.5 years (range: 35-86 years). According to esophageal cancer staging standards presented in the 2009 UICC TNM 7th edition, 12 patients (3.7%) were diagnosed with upper thoracic esophageal cancer, 205 (63.3%) with middle thoracic esophageal cancer, and 107 (33%) with lower thoracic esophageal cancer. All patients underwent radical esophagectomy, 267 patients received chest/abdomen two-field lymphadenectomy, and 57 received neck/chest/abdomen three-field lymphadenectomy. The median follow-up time was 54 months (95% CI, 2.0-91.6 months).

Postoperative pathological analysis showed that 4 patients exhibited positive margins, while 320 had negative margins. Pathologic cellular differentiation level analysis identified 28 cases at G1, 231 cases at G2, and 62 cases at G3. Concerning pathological T stage assessment, 4 cases were at Tis (carcinoma *in situ*), 46 at T1, 27 staged at T2, 126 at T3, and 121 cases were at T4 stage. N stages were characterized as follows: N0, 187 cases, N1, 111 cases, N2, 18 cases, and N3, 8 cases. The median OS was 54 months (95% CI, 36.3-76.7 months), with a median follow-up time of 54 months (95% CI, 2.0-91.6 months).

The distribution of eight measured imaging parameters is presented in Table 1. Of the 324 patients in the study, 43 patients did not undergo barium esophagraphy at the hospital; therefore, we were unable to analyze images of barium ingestion in these patients.

**Table 1:** Patient characteristics (clinical factors and imaging factors) distribution and Cox regression analysis results of radiographic factors across included patients

Characteristics	Numbers of patients	95% CI		Median	Min	Max	P Value^*^
		Lower bound	Upper bound				
Largest long diameter (mm)		324	19.6	50.9	34.7	14.4	75.8	0.000
Largest short diameter (mm)		324	11.1	31.4	20.9	9	63	0.094
Barium lesion length (mm)		281	0	70	40	0	120	0.104
CT lesion length (mm)		324	10	110	50	0	187.5	0.003
Maximal lesion area (mm^2^)		324	196.1	1091.6	535.5	0	2347	0.012
Maximal curvature angle: esophagus with aorta		324	0	196.5	87	0	360	0.086
Maximal curvature angle: esophagus with trachea		324	0	113.9	0	0	194	0.334
Length of pericardium invaded (mm)		324	0	37.5	10	0	60	0.229
Sex	Female	239						0.010
	Male	85						
Age			35	86	56.5 (yr)			0.820
Adjuvant chemotherapy	Yes	93						0.586
	No	231						
Adjuvantradiotherapy	Yes	22						0.226
	No	302						
Primary location	Upper	12						0.499
	Middle	204						
	Lower	108						
Positive margins	Yes	4						1.000
	No	320						
Pathologic cellular differentiation level	G1	28						0.550
	G2	231						
	G3	62						
Type of lymph node dissection	2-field	267						0.194
	3-field	57						
PathologicalTstage	Tis+T1	50						0.000
	T2	27						
	T3	126						
	T4	121						
Pathological N stage	N0	187						0.000
	N+	137						

^*^As determined by Cox-regression analysis.

### Analysis of case variable effects on survival

As shown in Table 1, we performed Cox regression analysis to determine potential impacts of various individual population variables as well as radiographic factors on equation calculation across the included patients. Gender, largest long diameter, CT lesion length, maximal lesion area, pathological T stage and pathological N stage were determined to be of significant influence (Table 1). Other demographic factors such as age, administration of adjuvant radiotherapy and adjuvant chemotherapy, location of thoracic esophageal cancer (upper, middle and lower) and type of lymph node dissection did not significantly affect survival.

### Barium and CT lesion length analyses

Significant differences in lesion length measured following barium intake and CT-determined lesion length were determined. (T = 7.14, *P* < 0.001). Calculation of mean CT lesion length minus the mean esophageal barium lesion length measurement was 10.99 mm (95% CI 7.97-14.02mm).

### Survival analysis of surgical staging

#### Relationship between pathological T stage and survival

**Figure 3:**
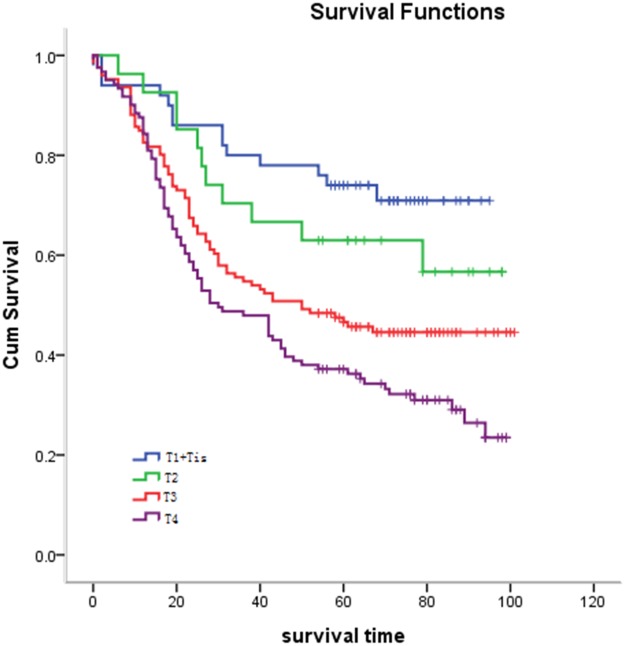
Relationship between pathological T stage and survival Statistical analysis revealed significant differences between pathological T stage and survival (*P* = 0.001).

The differences among different pathological T stages were statistically significant (*P* = 0.001). The resulting survival curve is shown in Figure 3. In staging groups, few patients were at Tis stage (*n* = 4) and they were therefore grouped with patients at T1. As such, all patients in T1 represent patients at Tis and T1 in the study.

#### Relationship between pathological N stage and survival

**Figure 4:**
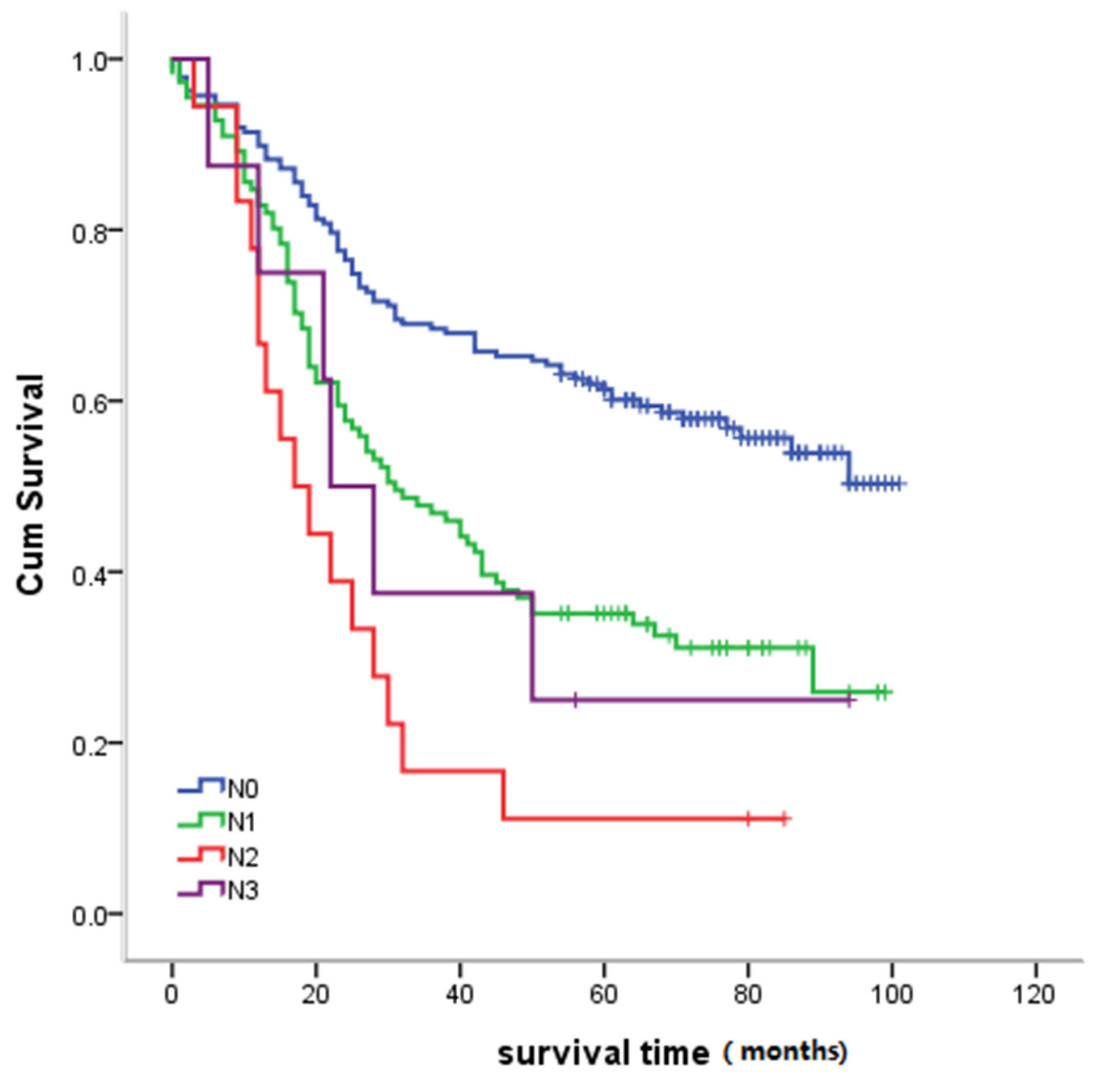
Relationship between survival and pathological N stage Statistical analysis revealed significant differences between survival and pathological N staging (*P* = 0.001).

As with T staging, survival differences between pathological N stages was statistically significant (*P* = 0.001) (Figure 4).

#### Relationship between histologic grade and survival time

**Figure 5:**
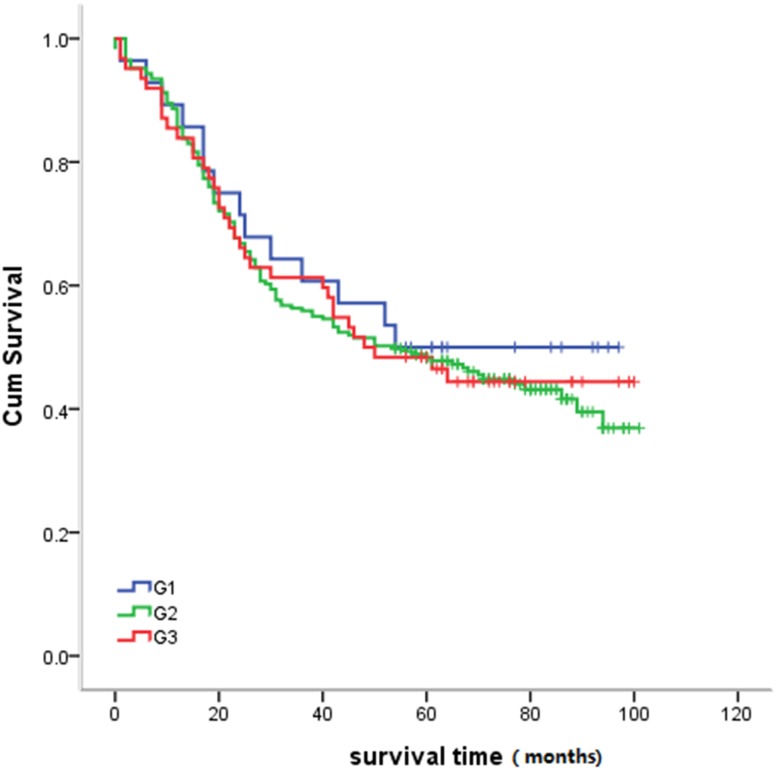
Relationship between histologic grade and survival Statistical analysis revealed no significant differences between histologic grade and survival (*P* = 0.85).

Tumor grade was not statistically significantly associated with survival time (*P* = 0.85). The survival curve is illustrated in Figure 5.

#### Grouping threshold for imaging factor and survival analysis

All cases were sorted according to the date of operation, with odd cases selected as a training set, and even cases used as validation sets. Clinical characteristics of patients in the training and validation sets are shown in Table 2. The clinical characteristics of patients in the two datasets are comparable. Using the method described in Equation (1), the measured values for each image factor in the training dataset was divided into four intervals and evaluated for significant differences in survival time among intervals in the validation dataset. The results show that through grouping maximum esophageal lesion area, largest long diameter and CT lesion length by appropriate intervals, there is a significant difference in the survival time for each factor (*p* < 0.05). Contrarily, for the factors of barium-indicated lesion length, the curvature formed by lesion contact with the trachea and thoracic aorta, and the length of pericardial fat reduction, there is no difference in the survival time through this grouping method. The optimal thresholds for grouping these indicators to reveal statistical differences in survival curves are discussed below. N0 and N1 were stratified according to lymph node status (because N2 and N3 were only 18 cases and 8 cases, respectively, and were grouped into the N1 category), and analysis of the relationship between imaging group and the survival time under different lymph node status was performed.

**Table 2:** Clinical characteristics of patients in training set and validation set

		Training set	Validation set	P Value
Age		57.46 (95%CI,55.98-58.85)	57.21 (95%CI,55.92-58.40)	0.19
Gender	Male	115	124	0.26
	Female	47	38	
pT	T1	27	23	0.92
	T2	13	14	
	T3	61	65	
	T4	61	60	
pN	N0	100	87	0.19
	N1	53	58	
	N2	5	13	
	N3	4	4	
G	G1	18	13	0.46
	G2	109	121	
	G3	33	30	

#### Groups based on maximum esophageal lesion area and survival

**Figure 6:**
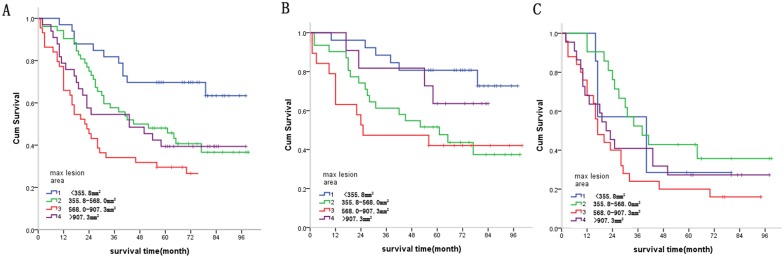
**(A)** Relationship between maximum esophageal lesion area and survival. Statistical analysis revealed significant differences between maximum esophageal lesion area and survival. (*X*^2^ = 15.862, *P* = 0.001). **(B)** Survival in pathological N negative patients was statistically correlated with maximum esophageal lesion area (*X^2^* = 10.138, *P* = 0.017). **(C)** Survival in pathological N positive patients was not correlated with maximum esophageal lesion area (*X^2^* = 4.924, *P* = 0.177).

As stated in Equation (1), a specified grouping method was utilized to achieve maximal survival curve differences between groups. A genetic optimization algorithm was applied to obtain the four major diameter groups: less than 355.8mm^2^, 355.8-568.0mm^2^, 568.0-907.3mm^2^ and greater than 907.3mm^2^. According to this threshold, the validation data sets patients were divided into four groups, image T1(iT1), image T2(iT2), image T3(iT3) and image T4(iT4), respectively. The patient number in each group and in N-state stratification is shown in Table 3. Survival was statistically correlated with maximum esophageal lesion area (*X*^2^ = 15.862, *P* = 0.001) (Figure 6A). Pathological N negative group: Survival was statistically correlated with maximum esophageal lesion area (*X*^2^ = 10.138, *P* = 0.017) (Figure 6B). Pathological N positive group: Survival was not correlated with maximum esophageal lesion area (*X*^2^ = 4.924, *P* = 0.177) (Figure 6C).

**Table 3:** The patient number of validation datasets in each group by radiographic factors based stage and the N state based stratification, correlation analysis between imaging group and survival

Radiographic factors based stage		iT1	iT2	iT3	iT4	P value
Maximum esophageal lesion area	pN0	26	31	19	11	0.017
	^*^N+	7	21	25	22	0.177
	Total	33	52	44	33	0.001
Largest long diameter	pN0	31	20	15	21	0.027
	^*^N+	10	14	24	27	0.027
	Total	41	34	39	48	0.001
CT lesion length	pN0	31	26	20	10	0.270
	^*^N+	12	17	21	25	0.224
	Total	43	43	41	35	0.048

NOTE: 1.^*^N+ includes pN1,pN2,pN3; 2. P value refer to correlation between radiographic factors based stage and the N state based stratification and survival.

#### Groups based on largest long diameter and survival

**Figure 7:**
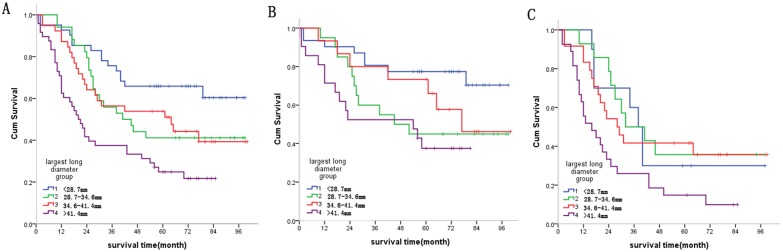
**(A)** Relationship between largest long diameter and survival. Statistical analysis revealed significant differences between largest long diameter and survival (*X^2^* = 20.810, *P* = 0.001). **(B)** Survival in pathological N negative patients was statistically correlated with largest long diameter (*X^2^* = 9.201, *P* = 0.027); **(C)** Survival in pathological N positive patients was statistically correlated with largest long diameter (*X^2^* = 9.246, *P* = 0.027).

Four largest long diameter groups, less than 28.7, 28.7-34.6mm, 34.6-41.4mm and greater than 41.4mm were established based on thresholds indicated by a genetic optimization algorithm. According to these thresholds, validation data set patients were divided into groups iT1, iT2, iT3 and iT4, respectively, Patient numbers in each group and in N-state stratification is shown in Table 3. Statistically significant differences in survival were identified between largest long diameter groups (*X*^2^ = 20.810, *P* = 0.001) (Figure 7A). For pathological N negative and pathological N positive groups, survival was statistically correlated with *largest long diameter* (*X*^2^ = 9.201, *P* = 0.027; *X*^2^ = 9.246, *P* = 0.027, respectively) (Figure 7B–7C, respectively).

#### Groups based on CT lesion length and survival curves

**Figure 8:**
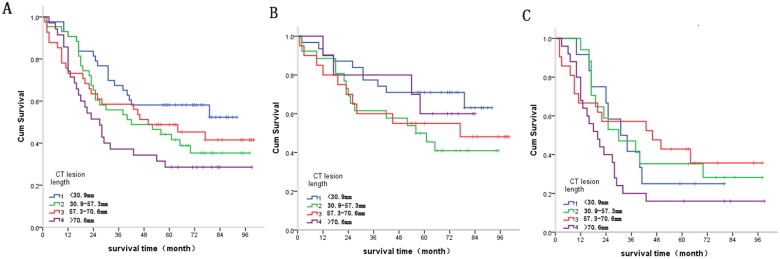
**(A)** Relationship between CT lesion length and survival. Statistical analysis revealed significant differences between CT lesion length and survival (*X*^2^ = 7.898, *P* = 0.048). **(B)** Survival in pathological N negative patients was not correlated with CT lesion length (*X*^2^ = 3.918, *P* = 0.270). **(C)** Survival in pathological N positive patients was not correlated with CT lesion length (*X^2^* = 4.374, P = 0.224).

A genetic optimization algorithm was applied to solve Equation (1) to generate four major groups based on lesion length indicated in CT imaging, less than 30.9mm, 30.9-57.3mm, 57.3-70.6mm and greater than 70.6mm. These lesion length-based validation data sets were designated groups iT1, iT2, iT3 and iT4, respectively. The patient number in each group and in N-state stratification is shown in Table 3. The CT lesion length groups were significantly different (*X*^2^ = 7.898, *P* = 0.048) (Figure 8A). For pathological N negative and pathological N positive groups, survival was not correlated with *CT lesion length* (*X*^2^ =3.918, *P* = 0.270; *X*^2^ = 4.374, *P* = 0.224, respectively) (Figure 8B–8C, respectively).

### Consistency analysis of pathological T staging and diameter based groups

The consistency between pathological T stage and maximum esophageal lesion area group, largest long diameter group and CT lesion length group are shown in Table 4 (*K* = 0.088, 0.118 and, 0.113, respectively). All diameter based groups and pathological T stage were not consistent.

**Table 4:** Consistency analysis of pathological T staging and diameter based groups

	Pathological stage					Total	KAPPA value
		1/Tis	2	3	4		
Max lesion area groups	1	32	10	16	13	71	0.088
	2	13	9	48	36	106	
	3	4	6	35	50	95	
	4	1	2	27	22	52	
Largest long diameter groups	1	34	9	20	18	81	0.118
	2	7	9	42	22	80	
	3	6	6	27	41	80	
	4	3	3	37	40	83	
CT lesion length groups	1	36	8	25	23	92	0.113
	2	8	6	43	36	93	
	3	4	10	29	30	73	
	4	2	3	29	32	66	
	Total	50	27	126	121	324	

### Survival analysis of diameter based groups across the different treatment

Survival analysis according to administration of adjuvant chemotherapy, adjuvant radiotherapy and type of lymph node dissection was performed on the three diameter based groups (Tables 5–7). No significant differences were observed in 3 field lymph node dissection and postoperative radiotherapy due to rare patient number. In postoperative adjuvant chemotherapy, patient survival in the iT4 group of the largest long diameter was superior to those without adjuvant chemotherapy (*X*^2^ = 6.003, *P* = 0.014).

**Table 5:** Survival analysis of radiographic-grouping by adjuvant chemotherapy

Radiographic-grouping		Adjuvant chemotherapy (yes = 1, no = 2)	Patient number	Total	Chi-Square	P
Largest long diameter group	1	1	24	90	0.004	0.950
		2	66			
	2	1	23	84	0.138	0.711
		2	61			
	3	1	18	74	0.951	0.330
		2	56			
	4	1	27	76	6.003	0.014
		2	49			
CT lesion length group	1	1	25	97	0.300	0.584
		2	72			
	2	1	32	88	0.000	0.986
		2	56			
	3	1	16	73	2.500	0.114
		2	57			
	4	1	19	66	0.129	0.719
		2	47			
Max lesion area group	1	1	18	78	0.281	0.596
		2	60			
	2	1	24	89	0.129	0.719
		2	65			
	3	1	23	78	0.268	0.604
		2	55			
	4	1	27	79	2.012	0.156
		2	52			
	Total		324			

**Table 6:** Survival analysis of radiographic-grouping by adjuvant radiotherapy

Radiographic-grouping		Adjuvant radiotherapy (yes = 1, no = 2)	Patient number	Total	Chi-Square	P
Largest long diameter group	1	1	5	90	1.644	0.200
		2	85			
	2	1	10	84	2.171	0.141
		2	74			
	3	1	2	74	0.083	0.774
		2	72			
	4	1	5	76	1.327	0.249
		2	71			
CT lesion length group	1	1	8	97	0.358	0.550
		2	89			
	2	1	9	88	3.415	0.065
		2	79			
	3	1	3	73	0.001	0.980
		2	70			
	4	1	2	66	0.606	0.436
		2	64			
Max lesion area group	1	1	8	78	1.196	0.274
		2	70			
	2	1	6	89	0.709	0.400
		2	83			
	3	1	3	78	8.928	0.003
		2	75			
	4	1	5	79	0.894	0.344
		2	74			
	Total	324				

**Table 7:** Survival analysis of radiographic-grouping by the type of lymph node dissection

Radiographic-grouping		Lymph node dissection type	Patient number	Total	Chi-square	P
Largest long diameter group	1	2	65	90	2.936	.087
		3	25			
	2	2	72	84	.376	.540
		3	12			
	3	2	63	74	1.170	.279
		3	11			
	4	2	67	76	.059	.808
		3	9			
CT lesion length group	1	2	74	97	6.567	.010
		3	23			
	2	2	70	88	.426	.514
		3	18			
	3	2	64	73	.002	.966
		3	9			
	4	2	59	66	8.377	.004
		3	7			
Max lesion area group	1	2	55	78	2.631	.105
		3	23			
	2	2	77	89	.292	.589
		3	12			
	3	2	66	78	.010	.920
		3	12			
	4	2	69	79	.925	.336
		3	10			
		Overall	324			

## DISCUSSION

Surgical and radiation therapies are primary treatment modalities for esophageal cancer. Precise staging prior to beginning therapy is critical for the development of individualized treatment programs and improves treatment efficiency and prognostic accuracy. Of all imaging techniques used for the purposes of esophageal tumor staging, the most common method is CT scanning. Other imaging technologies, including magnetic resonance imaging (MRI), endoscopic ultrasound (EUS), and positron emission (PET) scanning, are used increasingly frequently over time for clinical pre-treatment staging. Although the value of CT in clinical staging with use of barium tablets is somewhat inferior to MRI and EUS, the widespread use of CT and its resulting electron density data make CT desirable to many clinicians, especially radiation oncologists in particular, who utilize CT for 3-dimensional treatment planning.

The TNM staging of esophageal cancer, as stated in the current 7^th^ edition of AJCC/UICC, does not help determine clinical staging of esophageal cancer. Rice et al. [14] most recently reported that thirty-three institutions from six continents submitted data using variables with standard definitions: demographics, comorbidities, clinical cancer categories, and all-causes of mortality from first management decision. These data will form the foundation for the 8th edition cancer staging manuals following risk adjustment for patient characteristics, cancer categories, and treatment characteristics and should direct 9th edition data collection. However, it should be noted that Rice et al. concluded “it became evident that clinical categories did not share the same prognostic implications as pathologic categories after esophagectomy alone” [14]. In the study presented here, we compared imaging-based groups and pathological T staging and found no consistencies between imaging T groups and pathologic T staging. Therefore, it is necessary to establish a new clinical T staging approach based on imaging characteristics that are independent of pathological T staging. This new staging system may be valuable for clinical stage of non-surgical patients. Exploration of CT and other radiological imaging-based non-surgical staging still has practical value.

CT exhibits high density and spatial resolution and is very effective at demarcating esophageal borders and illustrating its association with surrounding tissues and organs. Currently CT scan imaging is the main foundation of esophageal gross tumor volume (GTV) delineation [15]. The thickness of normal esophageal wall varies due to different degrees of expansion; however, the wall thickness is usually less than 3mm. An esophageal wall thickness greater than 5 mm is usually regarded as abnormal, but unfortunately, CT based measurements of esophageal wall thickness are currently not very accurate. Studies have previously reported that the accuracy of CT diagnosis of esophageal cancer via T staging was 45% to 80% [16, 17], especially for early stage esophageal lesions, for example those confined to the mucosa or submucosa. During these early stages of disease, wall thickening is not obvious in CT images. The diagnostic accuracy of CT imaging in T staging is remarkably low, with accuracy rates reported as low as 33% [17, 18]. The esophageal mucosa imaging by barium swallow can display small changes, so for early esophageal lesions positioning and measurement. The combination of CT and X-ray imaging comprise a more accurate approach. Non-surgical staging of esophageal cancer is currently based on both CT, barium imaging and EUS (to determine depth of invasion), although it is likely that the eight indicators identified contribute differently by imaging type to non-surgical staging. It is also possible that some of these indicators could be substituted or replaced by others in particular types of assessment of association.

Various characteristics of tumors can be visualized using CT scanning in patients with esophageal carcinoma, such as tumor texture and other morphologic characteristics [19]. Of such characteristics, tumor length has extensive evidence supporting it as a characteristic linked to diagnosis and prognosis in ESCC [20–26]. In the present study, the measured CT esophageal lesion length is larger than barium lesion length, with a mean CT measured length of 10.99mm. Though barium swallow esophagram is often used as a primary imaging modality to identify esophageal masses and wall defects, it is of limited value for the purposes of TNM staging [27]. Concerning non-surgical staging, only one of two possible indicators can be utilized. Our conclusion that the CT-determined esophageal lesion length is greater than that determined via barium esophagram differs from the conclusions presented by Thompson et al [28]. In their study, they report that CT imaging often underestimates tumor length (up to 3cm) compared to imaging with barium swallow intake. Reasons for this difference may be a result of differences in diagnostic criteria. For example, Thompson et al. report that the standard measure of CT lesion length utilized was determined following lesion confirmation by three or more radiologists. Our study did not utilize these criteria in our assessments. In addition, our barium esophagrams may have actually underestimated the length of the tumors, contrary to what Thompson et al. observed. In studies using double-contrast barium imaging, early ESCC exhibits the appearance of small, polypoid lesions, or as flat lesions optimally visualized in profile [29, 30]. They could also appear as superficial mucosal nodules, which would be hard to quantify morphologically [29, 30]. On the opposite spectrum, large masses may have extensive spread and can be better visualized and measured with higher resolution CT imaging. Such underestimation could be why our barium-swallow esophagram measurements did not correlate with overall patient survival.

The present study identified a significant influence of gender on survival. In general, men are at least 3 times more likely to be diagnosed with esophageal cancer than women [31, 32]. Regarding different survival prognosis based different gender, women have a better prognosis upon diagnosis and treatment than men [33]. There are several possible reasons for these differences. Prevalence of ESCC is dependent on many factors including geographic location, lifestyle choices, nutritional quality, and temperature of foods and beverages have been suggested to be risk factors [34–38]. Of those that could account for differences in prevalence and poor prognosis in men, men are more likely to be involved in lifestyle activities such as tobacco and alcohol use [39–41], which are the most common risk factors for ESCC [37, 38]. For example, recent estimates in China for the percentage of males who smoke are about 50%, versus about only 3% of women [39].

Although histopathologic confirmation of malignancy by endoscopic biopsy and CT examination with intravenous contrast are primary and complimentary approaches to diagnosis and staging of esophageal carcinomas, consistency in staging between these modalities is surprisingly debatable. Extensive documentation and comparison of these techniques is available in the literature, and will not be discussed in further detail here. Rather, our focus was to highlight our novel approach to CT-based tumor staging and directly analyze relationships between radiological factors and survival time. By grouping tumor severity by CT and application of algorithms, we evaluated the influence of various different parameters on survival. In our study, we found significant correlations between survival and radiographic characteristics in accordance with algorithmic grouping. With grouping by genetic algorithm, survival time was significantly different among different intervals of CT imaging characteristics including maximum lesion area, largest long and short tumor diameter, and lesion length. The results of our analysis also indicate that surgical staging (T staging and N staging) correlated with survival, while tumor histologic grade was not associated with survival. Our findings highlight and further support the importance of CT imaging for ESCC lesion staging and diagnosis. In the max long diameter group of iT4 patients, the survival of patients with adjuvant chemotherapy was superior to those without adjuvant chemotherapy. In combination with algorithmic grouping, our study may contribute to the establishment of a novel sensitive CT image based clinical staging system, which is a technique that is widely applied clinically in China and other developing countries.

**Figure 9:**
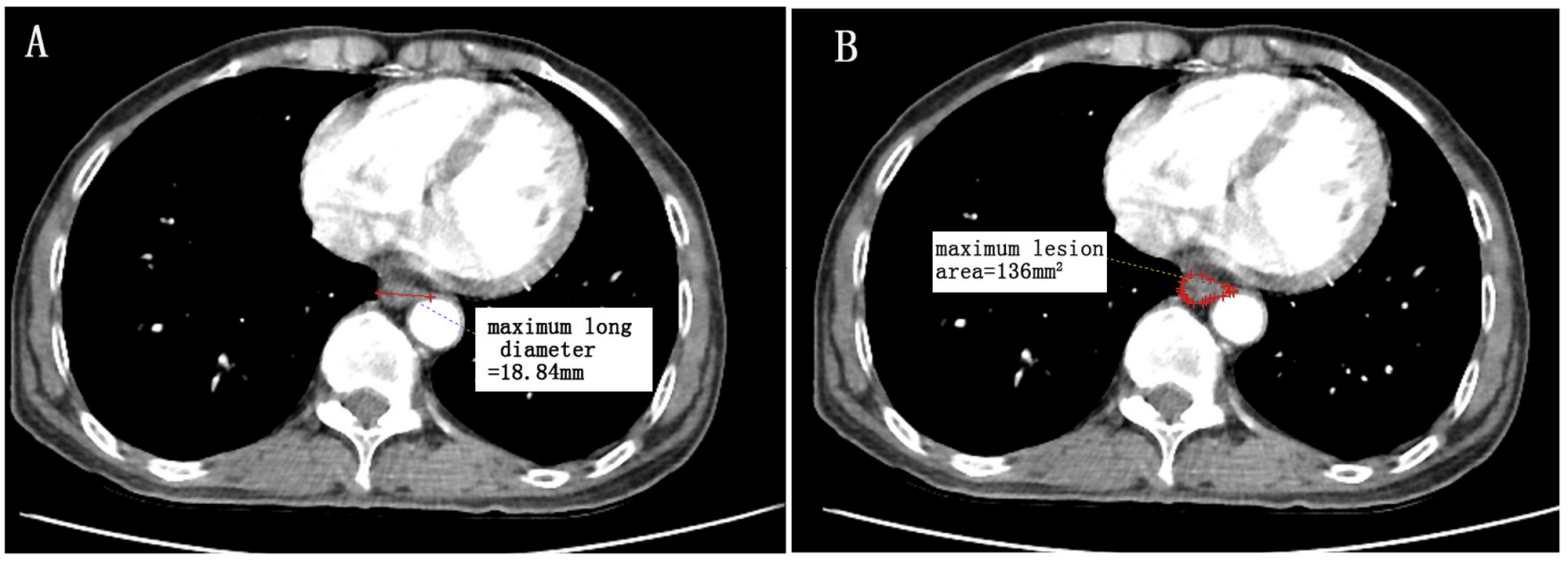
A patient with lower thoracic esophagus squamous cell carcinoma (T1N0M0) An illustration of radiographic factors and endoscopic pathology reports that the distance from the incisors to the proximal edge of the tumor is 36cm. **(A)** maximum long diameter (18.84mm), **(B)** maximum lesion area (136mm^2^). Since no wall thickness was greater than the 5mm level as determined by CT based diagnostic criteria, CT-based lesion length is considered to be 0mm. This patient is “iT1”. (Measured by Neusoft PACS/RIS version 3.1,Neusoft Beyond Technology).

In consideration of the effect of Tis, T1 and positive margin patients on prognosis and the existence of data measurement difficulties, it remains arguable whether to retain this part of the data. Several reasons exist that lead us to believe it is worthwhile to keep this data in the analysis. Firstly, when we exclude the Tis, T1 and positive margin patients and reanalyzed the data and performed a new comparison between the new and previous grouping thresholds (Table 8), we still identify a similar threshold for iT2iT3iT4 to that reported in our previous results. Secondly, the purpose of this study is to investigate radiographic factors as prognostic indicators for survival in ESCC and determine the appropriate radiographic parameters for clinic staging. Tis, T1 and positive margin identification is available only after pathological T staging is performed, although clinic staging typically occurs prior to pathological T staging. It is not possible to obtain this information during clinic staging. According to the Precision Medicine Core of the AJCC, “All predictors must be known at time zero” [42]. We suggest including these data to increase clinical practicability. Thirdly, another arguable point concerns the methods by which researchers delineate the lesions for Tis and T1 patients. We have described the possibility of measurement in our schematic drawing Figure 9. Therefore, we think it is necessary to include these patients in this study.

**Table 8:** Optimal thresholds for imaging in the total dataset and excluding patients with T1, Tis, and positive margins

	Maximum esophageal lesion area (mm^2^)		Largest long diameter (mm)		CT lesion length (mm)	
	All^*^	Delete^**^	All^*^	Delete^**^	All^*^	Delete^**^
iT1	<355.8	None	<28.7	None	<30.9	None
iT2	355.8-568.0	<548.4	28.7-34.6	<32.7	30.9-57.3	<60.1
iT3	568.0-907.3	548.4-960.5	34.6-41.4	32.7-40.3	57.3-70.6	60.1-71.8
iT4	≥907.3-	≥960.5	≥41.4	≥40.3	≥70.6	≥71.8

NOTE: All^*^, total dataset; Delete^**^ exclude patients with T1,Tis, positive margin;

None: Patients with T1 and Tis was deleted.

Limitations of this study should be noted. First, we were still unable to combine different imaging parameters into a single criterion for direct application to clinical staging. In addition, this study did not calculate survival-associated groups based on lymph node imaging. The role of T and N staging in esophageal cancer is inseparable, so we try to hierarchically analyze different image T stage by N-state to test whether the image T stage is prognostic in different N states, Although the combination of pathological N stage and image T stage is not related to any practical meaning. Only the largest long diameter was associated with survival in different N states. The maximum esophageal lesion area according to the image T stage are survival-related only in N-negative patients. CT lesion length image T stage did not correlate with survival after stratification by N state. This result may be because the CT image cannot clearly distinguish between esophageal lesions and esophageal lymph nodes. When we measure the largest long diameter, esophageal lymph nodes are inadvertently included, so that the largest long diameter can be associated with survival regardless of N status. According to the AJCC staging system, tumor length may be a strong surrogate benchmark for the presence or absence of nodal disease in early to intermediate stage esophageal cancer [43]. This maybe the reason why CT lesion length image T stage did not correlate with survival after stratification by N stage. Future research will address these limitations and build on the foundation the present study has provided. Continuation of this study will include additional focus on 1) the relationships between survival and imaging characteristics of thoracic esophageal cancer patients that underwent radical radiotherapy and chemotherapy; 2) the relationships between survival and the image-based nodal status (including lymph node status as shown by PET / CT).

## CONCLUSION

In conclusion, this is the first clinical study, to the best of our knowledge, to utilize a genetic algorithm to group various types of radiographic characteristics. We found that reasonable stratification of imaging factors, including maximum esophageal lesion area, largest long diameter and lesion length measured in CT is valuable for clinical T staging of thoracic esophagus squamous cell carcinoma. Further optimization and feasibility of this staging approach for patients with non-surgical treatments remains to be further validated.

## DECLARATIONS

### Ethics statement

Study participants voluntarily agreed to participate in the study and provided written informed consent prior to enrollment. The study was approved by the Ethics Committee of First Hospital of Quanzhou Affiliated to Fujian Medical University. All procedures performed in studies involving human participants were in accordance with the ethical standards of the institutional and/or national research committee and with the 1964 Helsinki declaration and its later amendments or comparable ethical standards.

### Consent for publication

All patients have agreed to use their information in this publication.

### Availability of data and material

All data generated during the project will be made freely available via First Hospital of Quanzhou Affiliated to Fujian Medical University’s Research Data Repository. DOIs to these data will be provided (as part of the DataCite programme) and cited in any published articles using these data and any other data generated in the project. There are no security, licensing, or ethical issues related to these data.
